# Developmental Coordination Disorder in a Patient with Mental Disability and a Mild Phenotype Carrying Terminal 6q26-qter Deletion

**DOI:** 10.3389/fgene.2017.00206

**Published:** 2017-12-06

**Authors:** Marianna De Cinque, Orazio Palumbo, Ermelinda Mazzucco, Antonella Simone, Pietro Palumbo, Renata Ciavatta, Giuliana Maria, Rosangela Ferese, Stefano Gambardella, Antonella Angiolillo, Massimo Carella, Silvio Garofalo

**Affiliations:** ^1^Dipartimento di Medicina e Scienze della Salute “V. Tiberio”, Università degli Studi del Molise, Campobasso, Italy; ^2^Unità Operativa di Medicina Trasfusionale, Azienda Sanitaria Regionale del Molise, Presidio Ospedaliero San Timoteo, Termoli, Italy; ^3^Unità Operativa Complessa di Genetica Medica, Poliambulatorio “Giovanni Paolo II”, IRCCS Casa Sollievo della Sofferenza, San Giovanni Rotondo, Italy; ^4^Ufficio per la Tutela della Salute Neurologica e Psichica dell’Età Evolutiva, Azienda Sanitaria Regionale del Molise, Termoli, Italy; ^5^IRCCS Neuromed, Pozzilli, Italy

**Keywords:** terminal 6q deletion, mental retardation, dyspraxia, Parkin, FRA6E

## Abstract

Terminal deletion of chromosome 6q is a rare chromosomal abnormality associated with variable phenotype spectrum. Although intellectual disability, facial dysmorphism, seizures and brain abnormalities are typical features of this syndrome, genotype–phenotype correlation needs to be better understood. We report the case of a 6-year-old Caucasian boy with a clinical diagnosis of intellectual disability, delayed language development and dyspraxia who carries an approximately 8 Mb *de novo* heterozygous microdeletion in the 6q26-q27 locus identified by karyotype and defined by high-resolution SNP-array analysis. This patient has no significant structural brain or other organ malformation, and he shows a very mild phenotype compared to similar 6q26-qter deletion. The patient phenotype also suggests that a dyspraxia susceptibility gene is located among the deleted genes.

## Case Presentation

Children with intellectual disability in the Molise (Italy) school district were enrolled in genetic screening for chromosomal defects and copy number variations (CNVs). This study was carried out following the recommendations of the Declaration of Helsinki and the Italian law for biomedical experimentation with written informed consent from all subjects. The protocol was approved and authorized by Italian National Health Service Azienda Sanitaria Regionale del Molise (ASREM) bio-ethical Committee with protocol no. 68 – November 5th, 2015 under the denomination “Epidemiologia delle malattie genetiche rare associate a sindromi dismorfiche e disabilità psichica nella Regione Molise.” All subjects or their legal guardians gave written informed consent under the Declaration of Helsinki and the approved protocol, including full consent to the publication of the case and related picture. Patients were carefully selected to undergo a preliminary cytogenetic screening followed by chromosome microarray analysis (CMA).

The patient was born from a 38-year old mother and a 40-year old father. He is the third son of non-consanguineous Caucasian parents and was born at 37 weeks of gestation by cesarean section. Birth weight was 2400 g (3rd centile), length 48 cm (21st centile) and head circumference 31.5 cm (3rd centile). The Apgar score was eight at 1 min and nine at 5 min.

The patient did not experience seizures, but his motor and language development was delayed. He started walking when he was 18 months old, and he pronounced the first words only around 3 years old. When he was 4 years old, the neuropsychological assessment showed developmental disorders of speech and language, hyperkinetic disorder, and global developmental delay.

**FIGURE 1:**
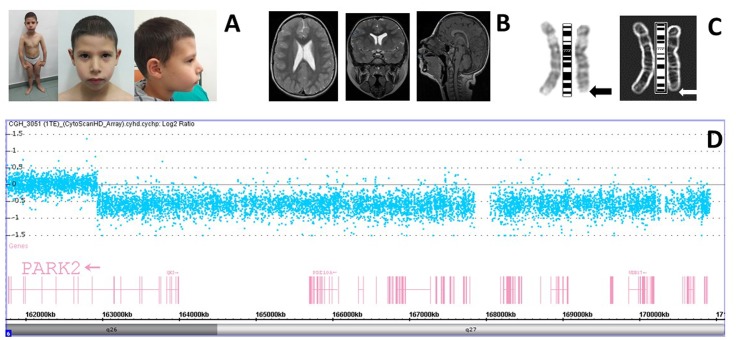
**(A)** The patient and his facies showing mild dysmorphological features. **(B)** Patient brain MRI is showing slight enlargement of the occipital horns with normal morphology. **(C)** Positive and negative views of the patient GTC-banded chromosomes six at 400 bands resolution with the arrows pointing to the deleted region. **(D)** High-resolution chromosome microarray analysis (CMA) in the patient. Deviations of probe Log2 ratios from zero of the SNP probes on chromosome 6q26-q27 cytoband indicate the deleted region identified in the genomic profile of the patient.

At the age of 6 years, the patient was referred to a clinical geneticist for evaluation of developmental delay and dysmorphic features. His physical examination showed microcephaly (head circumference, 48.5 cm, 1st centile), hypertelorism, depressed nasal root, bulbous tip, winged scapula, and toe walking (**Figure 1A**). He was clumsy in movements. Weight was 23 kg (72nd centile) and height was 117 cm (63rd centile).

Infant neuropsychiatric evaluation revealed mild cognitive delay (IQ of 67 using WPPSI-III), speech delay and learning difficulties, and developmental coordination disorder (DCD) (dyspraxia). The EEG examination showed the presence of minor electrical abnormalities (potential 4-5 c/s, 30–50 μV) prevailing on the left posterior regions. MRI of the brain detected slight enlargement of the ventricular system mainly in occipital horns with normal morphology (**Figure 1B**). Cardiac examination with echocardiogram was normal as well-ophthalmological and audiological evaluation. Ultrasound of kidneys and urinary tract shows the absence of anomalies. His medical history also included surgery to remove a thyroglossal duct cyst and frequent episodes of wetting and encopresis.

Chromosomes were obtained from cultured peripheral lymphocytes using Synchroset kit (Euroclone, Milano, Italy). The conventional technique of G banding analysis was used. DNA was extracted from whole blood using kit QIAamp DNA Mini (Qiagen, Hilden, Germany). DNA concentration and purity were determined with a ND-1000 Spectrophotometer (NanoDrop Technologies, Berlin, Germany).

The CytoScan HD array contains more than 2.6 million markers for the copy number analysis. Of these markers, 1,950,000 are unique, non-polymorphic oligonucleotide probes, and 750,000 are SNP probes used for genotyping. The average marker spacing is one probe per 1.1 kb, with a mean spacing of one probe per 1.7 kb on non-gene backbones and one probe per 880 bp in intragenic regions. The CytoScanHD assay was performed according to the manufacturer’s protocol, starting with 250 ng DNA. Briefly: total genomic DNA was digested with a restriction enzyme (NspI), ligated to an appropriate adapter for the enzyme, and subjected to PCR amplification using a single primer. After digestion, with DNase I, the PCR products were labeled with a biotinylated nucleotide analog, using terminal deoxynucleotidyl transferase and hybridized to the microarray. Hybridization was carried out in the Hybridization Oven 645 while subsequent washing and staining were performed using the Fluidics Station 450. The array was then scanned with the Scanner 3000 7G and both quality control step and copy number analysis were performed using the Chromosome Analysis Suite Software version 3.1. The clinical significance of each CNV detected was assessed by comparison with an internal database of 3,000 clinical samples and public database of CNVs (ISCA, ClinVar, DECIPHER, DGV). All nucleotide positions were based on the February 2009 human reference sequence, assembly GRCh37/hg19, produced by the Genome Reference Consortium.

Twenty metaphases of the patient and the parents were analyzed after GTG banding at 400 chromosome band resolution. Karyotype showed a small deletion in the terminal region of the long arm of chromosome 6 in all the analyzed metaphases. The karyotype of the child was 46, XY, del(6)(q26→qter) (**Figure 1C**). This rearrangement was not detected in the parents. To map deletion breakpoint more accurately high-resolution CMA was made.

Results of SNP array analysis of the patient displayed a copy number loss in chromosome bands 6q26-q27 of about 7,988 Mb ranging from nucleotide 162,931,432 to 170,919,482 (**Figure 1D**). In this interval there are 36 protein-coding genes: PARK2, PACRG, QKI, C6orf118, PDE10A, SDIM1, TFT, PRR18, SFT2D1, MPC1, RPS6KA2, RNASET2, FGFR10P, CCR6, GPR31, TCP10L2, UNC93A, TTLL2, TCP10, C6orf123, MLLT4, KIF25, FRMD1, DACT2, SMOC2, THBS2, WDR27, C6orf120, PHF10, TCTE3, ERMARD, DLL1, FAM120B, PSMB1, TBP, PDCD2 (according to DECIPHER v9.15 https://decipher.sanger.ac.uk/). The haploinsufficiency of some genes must be implicated in the clinical phenotype of this patient with the telomeric deletion of 6q26-q27.

According to the International System for Human Cytogenetic Nomenclature [ISCN, 2016], the molecular karyotype of the patient (showing both the minimum and maximum extent of the deletion) was defined as follows: arr[GRCh37] 6q26-q27(162,931,432-170,919,482)x1 dn. No other pathological CNVs were identified, excluding well-known benign CNVs variants.

The *PARK2* gene maps in 6q26 deletion breakpoint. Consequently, *PARK2* coding sequence (MIM number: 602544; NM_004562), exon/intron boundaries and flanking intronic regions, were analyzed by PCR and direct sequencing (Kitada et al., 1998) using ABI BigDye Terminator Sequencing Kit v.3.3 (Applied Biosystems, Foster City, CA, United States) and an ABI 310 Genetic Analyzer (Applied Biosystems). Sequencing did not show any other mutation.

## Background

About 1/2000 patients affected by mental retardation with developmental delay shows terminal deletion of the long arm of chromosome 6 (Lee et al., 2011). This condition is a rare cytogenetic disorder characterized by extended variation in the size of the deleted region that can go from cytogenetically visible to small submicroscopic deletions, ranging from 0.4 to 12 Mb (Lee et al., 2011). Only molecular cytogenetic techniques as fluorescent *in situ* hybridization (FISH) and comparative genomic hybridization (CGH) can precisely recognize and define the smaller terminal deletions (Erdel et al., 1997; Birnbacher et al., 2001; Sherr et al., 2005).

Patients with distal deletion of chromosome 6q show also variable phenotypic features with only a few common symptoms that include, besides intellectual disability and developmental delay (Bertini et al., 2006; Striano et al., 2006; Mosca et al., 2010), also hypotonic muscle, seizure (Elia et al., 2006; Striano et al., 2006), dysmorphic features with spinal cord and brain anomalies (Dupé et al., 2011; Nair et al., 2012). Agenesis/hypoplasia of the corpus callosum (Sherr et al., 2005), hydrocephalus (Li et al., 2015) or non-specific ventricle enlargement, lissencephaly, pachygyria, polymicrogyria, neuronal migration abnormality with periventricular nodular heterotopia (PNH) (Conti et al., 2013), olfactory bulb aplasia and anosmia (Gerber et al., 2011) are the most frequently described brain defects in these patients. Craniofacial anomalies (Hopkin et al., 1997) are also common, and they include hypertelorism with broad nasal bridge, midface hypoplasia with cleft palate, long philtrum, thin upper lip and ear anomalies. Also, heart defects (Nair et al., 2012), retinal abnormalities (Rivas et al., 1986; Abu-Amero et al., 2010), vertebral column defects (Li et al., 2015), joint laxity with elbow and knee anomalies, and attention deficit hyperactivity disorder (ADHD) are described in numerous patients.

Several authors have tried to correlate the distal deletion of chromosome 6q to a distinct clinical phenotype (Stevenson et al., 2004; Bertini et al., 2006; Rigon et al., 2011; Zhou et al., 2014). Others have sought to identify the minimum deleted interval containing the critical genes responsible for the major clinical problems (Eash et al., 2005; Rooms et al., 2006; Peddibhotla et al., 2015). However, it is still very problematic to identify clinically patients carrying such deletion and to correlate deleted genes with a clinical phenotype (Rigon et al., 2011).

## Discussion

Here, we report the clinical and genomic characterization of a patient with an intellectual disability, delayed language developmental and dyspraxia and minor dysmorphic features due to a non-recurrent 6q26-qter deletion identified using high-resolution CMA in a screening of patients with non-syndromic intellectual disability. In this patient haplodeficiency of chromosome 6q subtelomeric region is the result of a *de novo* event and it is not due to familiar unbalanced translocation. The breakpoint is mapped in 6q26 where is located the common fragile site FRA6E (**Figure 1D**). It is well-known that FRA6E is a genomic hotspot that predisposes to chromosomal deletion and it is considered the third most mutation-prone fragile site of the human genome (Palumbo et al., 2010; Ambroziak et al., 2015). In the center of this genomic site is located the PARK2 gene that is a huge gene implicated in the autosomal recessive early-onset type of Parkinson disease. The *PARK2* gene encodes the ubiquitin ligase Parkin that has high expression level in human neurons. We believe that *PARK2* haploinsufficiency may have a role in the etiology of intellectual disability of this patient since we can exclude mutations in the other *PARK2* allele. Micro-deletions or duplications and CNV in *PARK2* are found in patients with Autism Spectrum Disorder (Scheuerle and Wilson, 2011). In mouse, *Park2* knock out does not reproduce any sign of the Parkinson disease (Perez and Palmiter, 2005). Instead, Parkin null mouse and the *quaking* mutant mouse, who carries a 1,8 Mb deletion of mouse chromosome 17 including *Park2*, show mainly neurological and behavioral problems (Lorenzetti et al., 2004; Wilson et al., 2010).

The patient displays a cytogenetically visible deletion of about 8 Mb in size that causes haploinsufficiency of other 36 proteins coding genes. RNASET2, CCR6, DACT2, SMOC2, DLL1, and TBP are other brain-specific genes located in the deletion interval. They are all excellent candidate genes to affect brain functions and to generate with Parkin the clinical spectrum of the 6qter syndrome. However, the patient shows a mild phenotype. He has microcephaly, minimal enlargement of the cerebral ventricular system mainly in occipital horns and minor electrical abnormalities prevailing on the left posterior regions of the brain. Cerebral malformations are absent as eye and heart anomalies.

As described in previous studies (Stevenson et al., 2004; Bertini et al., 2006; Zhou et al., 2014), the 6q distal deletions are characterized by highly variable clinical symptoms that do not depend on the extension of the chromosomal abnormality. This condition can also happen in other contiguous gene deletion syndromes because any CNV can also affect the expression level of genes situated nearby the deleted or duplicated region, modulating the clinical consequences of haploinsufficiency (Stranger et al., 2007). This “position effect” can explain the complexity and heterogeneity of the phenotype associated with terminal 6q deletion (Stranger et al., 2007).

Peddibhotla et al. (2015) have reported a very similar 6q terminal deletion in a female newborn. She had a 7.6 Mb heterozygous deletion at 6q26-qter between nucleotides 162,784,565–170,899,992 which determined multiple organ anomalies (right-sided subependymal nodular gray matter heterotopia and agenesis of the corpus callosum, bicuspid aortic valve, imperforate anus with perineal fistula, segmentation anomalies of the sacrum, dextroconvex scoliosis of the lumbosacral spine and tethered spinal cord). None of these symptoms are present in this patient. However, both patients showed hypertelorism, broad nasal bridge, and global developmental delay. Correlation between the two patients indicates that terminal 6q deletions that are genotypically almost overlapping can be remarkably dissimilar in phenotype and clinical severity.

## Concluding Remarks

To our knowledge, this is the first case of association between terminal 6q deletion and DCD. However, there are several genes in 6q26-27 region. Studies may narrow down the DCD candidates in the near future.

## Author Contributions

MDC, SiG, RC, and GM were responsible for clinical data and sample collection. MDC, AS, EM, PP, and OP made DNA extraction, karyotype, and CMA. RF and StG Park2 sequencing. MDC, AA, MC, and SiG analyzed the data and wrote the manuscript.

## Conflict of Interest Statement

The authors declare that the research was conducted in the absence of any commercial or financial relationships that could be construed as a potential conflict of interest.
